# Editorial: CD1- and MR1-Restricted T Cells in Antimicrobial Immunity

**DOI:** 10.3389/fimmu.2015.00611

**Published:** 2015-12-02

**Authors:** S.M. Mansour Haeryfar, Thierry Mallevaey

**Affiliations:** ^1^Department of Microbiology and Immunology, Western University, London, ON, Canada; ^2^Division of Clinical Immunology and Allergy, Department of Medicine, Western University, London, ON, Canada; ^3^Centre for Human Immunology, Western University, London, ON, Canada; ^4^Lawson Health Research Institute, London, ON, Canada; ^5^Department of Immunology, University of Toronto, Toronto, ON, Canada

**Keywords:** CD1, MR1, NKT cell, MAIT cell, infection, inflammation, immunity, cytokine

The main function of the immune system is to protect the host against microbial pathogens and their deleterious products. Innate defense mechanisms quickly eliminate infectious intruders or keep them in check until highly specific adaptive responses that also give rise to immunological memory are launched. Major histocompatibility complex (MHC)-restricted T cells are key players of adaptive immunity. The remarkable diversity of their T cell receptors (TCRs) allows for specific recognition of peptides derived from virtually all protein antigens (Ags) including those harbored or even modified by the most vicious pathogens encountered over one’s lifetime. Conventional T cells sense and respond to pathogen-derived peptides complexed with polymorphic MHC molecules. This is called the rule of MHC restriction (1).

Recent years have witnessed a growing appreciation for effector and regulatory functions of innate-like T cells that are restricted by non-polymorphic, MHC-like molecules. These include CD1-restricted T cells [e.g., natural killer T (NKT) cells] and MR1-restricted mucosa-associated invariant T (MAIT) cells, which are the subjects of discussion by leading experts in this Research Topic. These “unconventional” T cells may directly target infected cells, but are best known for their ability to swiftly secrete T helper 1 (Th1)-, Th2-, and/or Th17-type cytokines very early in the course of immune responses. These cytokines in turn modulate the function of numerous cell types including NK cells, macrophages, dendritic cells, conventional CD4^+^ and CD8^+^ T cells and B cells, all of which play critical roles in innate or adaptive antimicrobial immunity.

CD1 molecules appeared around 300 million years ago. They display a high degree of conservation across vertebrates (2). However, considerable inter-species variation exists in terms of the number of CD1 isoforms expressed. In humans, the CD1 locus encodes five isoforms (i.e., CD1a–e), whereas rodents express only CD1d (3, 4). With the sole exception of CD1e, CD1 molecules are cell surface glycoproteins directly participating in lipid Ag presentation to T cells. CD1a–c, which comprise Group 1 CD1 molecules based on their genomic homology and location, have been a focus of many studies on host responses to *Mycobacterium tuberculosis* lipids (5). By contrast, certain self-lipids and exogenous glycolipids derived from a relatively wide spectrum of pathogens can be presented to NKT cells by CD1d, which is considered the Group 2 CD1 molecule. Positive selection of both type I and type II NKT cells in the thymus also requires their interaction with CD1d (6). These cell types are commonly referred to as invariant NKT (*i*NKT) and variant NKT (*v*NKT) cells, respectively. The discovery of *i*NKT cell restriction by CD1d and the ability of these cells to recognize α-galactosylceramide (α-GalCer) (7) prompted the invention of α-GalCer-loaded CD1d tetramer reagents (8, 9). This can be viewed as a technical breakthrough that has allowed for accurate identification and functional analysis of *i*NKT cells in mice and humans. Subsequently, CD1d tetramers loaded with the myelin-derived glycolipid sulfatide were generated and employed to identify a substantial fraction of *v*NKT cells (10). Of note, CD1d-restricted, sulfatide-reactive γδ T cells have also been described (11, 12). To what extent CD1d-restricted γδ T cells may contribute to the resolution of infection is not clearly understood. γδ T cells are not a major focus of this Topic, but have been introduced and briefly discussed (13, 14).

*i*NKT cells are perhaps the most widely studied population of CD1-restricted T cells. They are relatively infrequent in circulation and in most lymphoid and non-lymphoid tissues. However, they amass in the mouse liver and in the human omentum (15), which was dubbed the “abdominal policeman” in 1906 (16). *i*NKT cells express NK cell markers along with a canonical TCR consisting of an invariant α chain (Vα14-Jα18 in mice and Vα24-Jα18 in humans) and one of the only few β chain choices, namely mouse Vβ8.2, Vβ2, or Vβ7 and human Vβ11. Positive selection of *i*NKT cells is executed by CD1d^+^CD4^+^CD8^+^ thymocytes (6) However, endogenous CD1d ligand(s) involved in *i*NKT cells’ thymic selection have been elusive. KRN7000, an exogenous glycolipid superagonist of *i*NKT cells, was first extracted from the sea sponge *Agelas mauritanius* in a screen for novel antitumor compounds (7, 17). It has a unique α-GalCer structure and is likely to have originated from microbes forming a symbiotic relationship with *A. mauritanius*. Up until a short time ago, α-GalCer was considered unnatural to mammals. However, a recent study documented the presence of α-anomeric glycosylceramides including α-GalCer, in minute quantities, within mammalian cells, which could serve as endogenous *i*NKT cell Ags (18). α-GalCer has been utilized as a powerful experimental tool in many mouse studies and as a therapeutic agent in several clinical trials for cancer and viral diseases (19, 20).

*i*NKT cells are among first-line emergency responders to microbes. They quickly accumulate at the sites of infection, injury or inflammation to aid in mobilization and activation of other immune cells (21). When infection alters *i*NKT cell numbers within a given tissue, it is informative to distinguish between their recruitment into the tissue and their *in situ* expansion, retention or redistribution. Intravital imaging techniques have permitted the visualization and monitoring of *i*NKT cells’ behavior in live animals (22, 23). They have revealed, for instance, that shortly after infection with *Borrelia burgdorferi*, the causative agent of Lyme disease, hepatic mouse *i*NKT cells cease to crawl along the liver sinusoids, but instead form clusters and establish firm adhesion with Kupffer cells that have engulfed the blood-borne spirochete (24). By contrast, within the joints, extravascular *i*NKT cells are not stationary and move along blood vessel walls toward *B. burgdorferi* (25). This is followed by a direct interaction with the bacterium and its elimination. There currently exists an unmet need to track and quantify CD1d-mediated presentation of pathogen-derived lipids and to investigate the characteristics of the immunological synapses formed between CD1d^+^ Ag-presenting cells (APCs) and *i*NKT cells during infection. As such, antibodies to CD1d:glycolipid complexes and soluble *i*NKT cell TCR reagents similar to those engineered before (26, 27) may prove valuable.

Lipid Ags that can be bound to CD1d and directly detected by *i*NKT cells are present in a number of pathogenic or commensal bacteria or protozoan parasites (21, 28, 29). *Sphingomonas* spp., *Bacteroides fragilis*, *B. burgdorferi*, *Helicobacter pylori, Streptococcus pneumoniae*, *Streptococcus agalactiae*, *Leishmania donovani* and *Entamoeba histolytica* are examples of such microorganisms. Viruses do not possess lipid ligands for *i*NKT cell TCRs. However, infection with some viruses (e.g., dengue virus) leads to upregulated CD1d expression consistent with *i*NKT cell activation while certain others (e.g., herpesviruses and HIV) downregulate CD1d to plausibly evade detection by *i*NKT cells (30, 31). Viral infection may also induce a shift in host cell lipid profiles, thus yielding more “antigenic” CD1d ligands (32). The presence of CD1d:self-lipid complexes is also often required for cytokine-mediated stimulation of *i*NKT cells during bacterial and viral infections. This typically occurs shortly after microbial components engage APCs’ Toll-like receptors and induce interleukin (IL)-12 and IL-18 secretion (13, 33). Finally, *i*NKT cells can be activated in a truly CD1d-independent fashion, for instance by a combination of IL-12 and IL-18 (34) or by group II superantigens of staphylococcal and streptococcal origin (35, 36).

Both protective and pathogenic roles for *i*NKT cells have been reported in infectious disease models. Perhaps even more intriguing, *i*NKT cell activation could have seemingly contradictory consequences during infection with different species of the same pathogen (e.g., *Chlamydia* spp.) (37). CD1d^−/−^ and Jα18^−/−^ mice have been used extensively to address the contributions of *i*NKT cells in infection and immunity. It is noteworthy that CD1d^−/−^ mice are devoid of not only *i*NKT cells but also *v*NKT cells. Moreover, Bedel et al. found that the cellular deficiency of the original Jα18^−/−^ mice is more broad than initially thought and that the TCRα repertoire in these mice is shrunk by ~60% (38). Therefore, except in cases where CD1d^−/−^ or Jα18^−/−^ mice have been reconstituted with *i*NKT cells as a confirmatory measure, it may be necessary to revisit earlier findings in these animals. This is now possible thanks to the recent development of a new version of Jα18^−/−^ mice with an exclusive *i*NKT cell deficiency (39) and a monoclonal antibody that can selectively deplete *i*NKT cells *in vivo* (40).

The role of *i*NKT cells in infection is dictated, at least partially, by the pro- versus anti-inflammatory dominance of the cytokines they produce. For instance, interferon (IFN)-γ-secreting *i*NKT cells contribute to the immunopathology of sepsis in the aftermath of polymicrobial infection, which can be therapeutically attenuated by Th2-skewing glycolipids (41, 42). α-GalCer analogs with Th1-polarizing properties have also been synthesized and may be considered as adjuvant candidates in preventative vaccination (43) and in immunotherapy of infectious diseases that can be potentially resolved by Th1-dominant responses (44).

*v*NKT cells, as indicated by their name, have a relatively heterogeneous αβ TCR repertoire (45). They are present in mice but more prominent in humans (14, 46). Unlike *i*NKT cells, *v*NKT cells are unresponsive to α-GalCer. A large sub-population of *v*NKT cells react with sulfatide, a self-glycolipid that is abundant within the central nervous system, liver, kidney, and pancreas. Several endogenous lipids other than sulfatide have been found to activate *v*NKT cells, suggesting that *v*NKT cells’ recognition mode can be both specific and somewhat promiscuous. Therefore, it is not unreasonable to assume that self-lipids released from infected or damaged cells or even microbial lipids cross-reactive with self-components may be presented by CD1d to elicit *v*NKT cell responses. Potent immunomodulatory cytokines secreted by *v*NKT cells mediate protection from or pathology associated with infection (14, 46). They also enable *v*NKT cells to establish cross-talk with other cell types including but not limited to *i*NKT cells. In fact, *v*NKT and *i*NKT cells may fulfill opposing functions during infection, as exemplified by parasitic infections of mice with *Trypanosoma cruzi* (47) and *Schistosoma mansoni* (48). The limited availability of reliable reagents and tools for *v*NKT cell studies constitutes a hurdle in delineating the significance of these cells in infectious diseases (14). Sulfatide-loaded CD1d tetramers are not sufficiently stable nor do they stain all *v*NKT cells. The advent of 24αβ mice, a transgenic mouse line carrying the rearranged Vα3.2/Vβ9 TCR of a *v*NKT cell hybridoma, shed light on certain aspects of *v*NKT cell biology and development (49). More recently, Jα18-deficient, IL-4 reporter (Jα18^−/−^4get) mice were employed to characterize *v*NKT cells at a polyclonal level (50). These mice were found to be responsive to multiple lipid Ags but not to sulfatide and several phospholipids. Therefore, until new models become available, we will continue to depend on these models and on parallel examination of CD1d^−/−^ and the exclusively *i*NKT cell-deficient version of Jα18^−/−^ mice to study *v*NKT cell responses *in vivo*.

Mucosa-associated invariant T cells are one of the hottest topics in immunology today (51, 52). They express an invariant TCRα chain (mouse Vα19-Jα33 and human Vα7.2-Jα33) (53, 54), and undergo positive selection by MR1^+^CD4^+^CD8^+^ thymocytes (55). Similar to CD1, MR1 is conserved among diverse mammalian species. MAIT cells preferentially home to mucosal tissues – hence their denomination. In human, they circulate at high frequencies in the blood and also make up ~50% of the entire hepatic T cell population. MAIT cells are absent from the peripheral tissues of germ-free mice (55), indicating a strict requirement for commensal microflora in MAIT cell homeostasis. It has been hypothesized that gut dysbiosis in diseased states (e.g., type 1 and type 2 diabetes) may change MAIT cell frequencies and functions with metabolic and inflammatory repercussions (29). MAIT cells are rare in wild-type mice, and Vα19 transgenic mice were generated to circumvent the feasibility limitations of mouse studies. Although several differences have been reported between mouse and human MAIT cell compartments, recent work suggests that MAIT cells from wild-type mice resemble their human counterparts more closely than previously appreciated (56).

MR1 tetramers loaded with reduced 6-hydroxymethyl-8-d-ribityllumazine, a MAIT cell Ag, were recently developed to enable positive identification of mouse and human MAIT cells (57). The vitamin B2 (riboflavin) biosynthesis pathway supplies MAIT cell ligands (58–60). Importantly, this pathway operates in microbes that activate MAIT cells, but not in mammals. However, host-derived metabolites may potentially form adducts with intermediates of the riboflavin pathway to generate MAIT cell neo-antigens (59). MR1 ligands are ubiquitous and harbored by many bacteria, including commensals. Therefore, how *in vivo* MAIT cell responses are controlled remains to be elucidated. Novel MR1 ligands that do not activate MAIT cells on their own but compete with bacterial and synthetic MAIT cell stimuli have been synthesized (60). This may inspire the development of MAIT cell inhibitors for experimental and therapeutic purposes.

MAIT cells can respond to numerous bacterial strains and yeasts (51, 52). These include *Klebsiella pneumoniae, Pseudomonas aeruginosa, Lactobacillus acidophilus, Staphylococcus aureus, Staphylococcus epidermidis*, *Candida glabrata*, *Candida albicans* and *Saccharomyces cerevisiae*. To test the *in vivo* significance of MAIT cells in anti-bacterial immunity, MR1^−/−^ mice have been utilized and shown to be unable to control infection with *K. pneumoniae*, *Mycobacterium bovis* bacillus Calmette–Guérin (BCG) or *Francisella tularensis* (61–63). Last but not the least, MAIT cells can be activated by a combination of IL-12 and IL-18 in an MR1-independent manner (64), which may be important for antiviral defense.

Innate-like T cells are fast-acting and occupy strategic locations in the body. Unlike classical MHC molecules, CD1 and MR1 exhibit limited polymorphism. Therefore, it is only fitting that CD1 and MR1 ligands are considered by many as attractive targets for vaccination of genetically diverse human populations. Despite gaps in our knowledge in this exciting area, which are outlined by experts in this Topic, the availability of powerful tools, reagents and models has fueled further interest in CD1- and MR1-restricted T lymphocytes. α-GalCer has been used in clinical trials, and Th1- and Th2-promoting, disease/infection-tailored glycolipid agonists of *i*NKT cells may find their way into clinical practice in the future. Furthermore, it is not too far-fetched to anticipate that once the role of MAIT cells in various infectious diseases is known, their manipulation by synthetic ligands and inhibitors can be achieved and potentially used in immunotherapeutic protocols. The time is ripe for both curiosity-driven and translational studies on CD1- and MR1-restricted T cells.

## Author Contributions

SMMH and TM served as co-editors for this Research Topic. SMMH wrote the Editorial.

## Conflict of Interest Statement

The authors declare that the research was conducted in the absence of any commercial or financial relationships that could be construed as a potential conflict of interest.
